# Efficacy and safety analysis of the use of ibrutinib associated with rituximab for the first-line treatment of patients with chronic lymphocytic leukaemia

**DOI:** 10.1016/j.htct.2025.106234

**Published:** 2025-12-15

**Authors:** Aline do Nascimento, Daniel da Silva Pereira Curado, Thais Montezuma, Wallace Breno Barbosa, Juliana Machado-Rugolo, Mariana Millan Fachi

**Affiliations:** a Universidade de Brasilia, Distrito Federal, Brazil; b Ministério da Saúde, Distrito Federal, Brazil; c Hospital Alemão Oswaldo Cruz, São Paulo, Brazil; d Hospital das Clínicas da Faculdade de Medicina de Botucatu, Botucatu, Brazil; e Universidade de Sorocaba, Sorocaba, Brazil

**Keywords:** Systematic review, Chronic lymphocytic leukaemia, Ibrutinib, Rituximab

## Abstract

**Introduction:**

Chronic lymphocytic leukaemia, a common blood cancer in adults, particularly affects the elderly and is marked by the accumulation of B lymphocytes. While therapeutic options have expanded, the fludarabine, cyclophosphamide, and rituximab (FCR) regimen remains the standard first-line treatment for fit patients in the Brazilian public health system.

**Aim:**

This systematic review aimed to assess the efficacy and safety of ibrutinib plus rituximab (IR) as a first-line therapy for chronic lymphocytic leukaemia.

**Methods:**

Following PRISMA guidelines and registered in PROSPERO (CRD42023494868), searches were conducted in multiple databases in December 2023 to identify relevant randomized controlled trials comparing the IR and FCR regimens. Eligible studies reported at least one of the following outcomes: progression-free survival, overall survival, severe adverse events, or quality of life.

**Results:**

Two double-blind randomized controlled trials (FLAIR and E1912) totalling 1300 patients met inclusion criteria. Meta-analysis showed that the IR regimen significantly improved progression-free survival compared to the FCR regimen (Hazard ratio: 0.41; 95% CI: 0.31–0.53) with moderate certainty of evidence. However, overall survival did not differ substantially (Hazard ratio: 0.71; 95% CI: 0.33–1.49), and the certainty of the evidence was very low. Quality of life data were unavailable. Due to variations in follow-up, results for severe adverse events were not pooled and the individual studies reported results with low certainty of evidence. The global risk of bias was rated as there was some concern due to the lack of concealed allocation in all outcomes.

**Conclusion:**

The IR regimen demonstrated superior progression-free survival and comparable safety to the FCR regimen suggesting it is an effective and safe option for first-line treatment of chronic lymphocytic leukaemia.

## Introduction

Chronic lymphocytic leukaemia (CLL) is one of the most common blood cancers in adults. It is most frequently diagnosed among people aged 65–74 (median age: 69) and is characterized by the proliferation and accumulation of small immunocompetent B lymphocytes in the peripheral blood, bone marrow, lymph nodes, and spleen [[1], [2], [3], [4], [5], [6], [7]].

Recent estimates indicate the incidence of CLL at 4.5 per 100,000 per year (male 5.8; female 3.3) [8], reaching 30 per 100,000 per year at an age greater than 80 years [4]. The death rate is 0.8 per 100,000 per year, and the 5-year relative survival rate is 88.1 % [8]. In Brazil, 11,540 cases of all types of leukaemia, myeloid or lymphocytic, acute or chronic, are expected per year between 2023–2025, corresponding to an estimated risk of 5.33 per 100,000 per year [9].

The International Workshop on Chronic Lymphocytic Leukaemia (iwCLL) guidelines define recommendations on how to establish the diagnosis of CLL and detailed description of the assessment of the treatment response [10]. Initially, LLC frequently tends to be asymptomatic and an isolated peripheral blood lymphocytosis [7]. Otherwise, the most common clinical presentation is lymphadenopathy, spectral B symptoms (i.e., fever, night sweats, weight loss, fatigue) or cytopenias (i.e., anaemia, thrombocytopenia, neutropenia) due to marrow infiltration, although with lower frequency [11]. The prognosis for CLL is variable [2,5,6]; while some patients have rapidly progressive courses and die soon after diagnosis, other patients survive for a long time and die from causes not related to CLL [5].

Therapeutic options for the treatment of CLL have expanded over time. The best option should be based on disease stage, presence or absence of del (17p) or *TP53* mutations, immunoglobulin heavy-chain variable region (*IGHV*) mutation status, patient age, performance status and comorbid conditions, and the agent’s toxicity profile. Fludarabine plus cyclophosphamide (chemotherapy) associated with rituximab (immunotherapy) regimens (FCR) remain first-line therapy due to their response rates and improved overall survival (OS) in specific subgroups of fit patients with previously untreated CLL. However, a continuous regimen with ibrutinib (targeted therapy) associated with rituximab (IR regimen) has also been considered an option in first-line treatment with improved efficacy and safety compared to the FCR regimen [12].

FCR is available in the Brazilian public health system (SUS) for the first-line treatment of CLL [13]. However, ibrutinib has recently been evaluated by the Commission for the Incorporation of Health Technologies into the SUS (Conitec) for the second-line treatment of CLL but was not recommended because of its incremental cost-effectiveness ratio [14]. However, Conitec has not yet evaluated IR as the first-line treatment of CLL. Thus, to identify the gaps that should be addressed, the present study aims to identify all peer-reviewed literature reporting the efficacy and safety of IR versus FCR in the first-line treatment of CLL patients.

## Material and methods

### Research strategy

The present systematic review was conducted according to the Preferred Reporting Items for Systematic Reviews and Meta-analyses (PRISMA) guidelines. The systematic review protocol is registered in the International Prospective Register of Systematic Reviews (PROSPERO) database under the number CRD42023494868. Searches were conducted in December 2023 in Medline (PubMed), EMBASE, Cochrane Library and ClinicalTrials.gov databases using indexed descriptors and a combination of free terms (Supplementary material –Tables S1 and S2). The research question was: is ibrutinib associated with rituximab (IR) more effective and safer than chemoimmunotherapy (FCR) for first-line treatment of CLL in patients?

Two investigators independently selected the articles, first reading the title and abstract, and then studies that met the inclusion criteria were included for a full-text review. After open-blinding, any discrepancies identified were resolved by the investigators through discussion and consensus. Excluded studies are listed in Supplementary material. The Rayyan software was used to optimize the selection [15].

### Inclusion and exclusion criteria

According to the PICO framework (Table S1), randomized controlled trials (RCTs) were eligible if they compared the IR with FCR regimens for the first-line treatment of naïve CLL patients. Eligible studies had to include at least one of the following outcomes: progression-free survival (PFS), OS, severe adverse events (SAE) or quality of life (QoL).

The search had no restrictions related to the year of publication, language of study, patient age, gender, ethnicity or presence of comorbidities. Results published as conference abstracts were excluded.

### Data extraction

After reading the full text, the data from eligible studies related to the author, year of publication, study design, inclusion and exclusion criteria, study location, number of participants, interventions, age, sex, follow-up, and efficacy and safety outcomes were extracted in a predefined Microsoft Office Excel spreadsheet.

### Analysis plan

The data were analysed qualitatively and were reported in tables. The effect size was presented by the hazard ratio (HR) and 95 % confidence interval (95 % CI) for survival outcomes, by the relative risk (RR) and 95 % CI for the outcome of SAE, and by the mean difference and 95 % CI for quality of life, if data outcomes were available in included studies.

When possible, a meta-analysis was performed using the random effects model employing the Review Manager software (version 5.4). The heterogeneity of the studies was verified by visual inspection of forest plots and by the Chi square (p-value <0.05) and I² values.

For outcomes with sufficient data, subgroup analyses were performed based on mutations.

### Risk of bias and certainty of evidence analysis

The risk of bias was evaluated using the Cochrane Risk of Bias (RoB 2.0) tool [16], and the certainty of the evidence was assessed with the Grading of Recommendations Assessment, Development and Evaluation (GRADE) tool [17]. Each assessment was conducted independently by two investigators, ensuring the highest level of objectivity and transparency. Any discrepancies were resolved through consensus.

## Results

**Figure 1:**
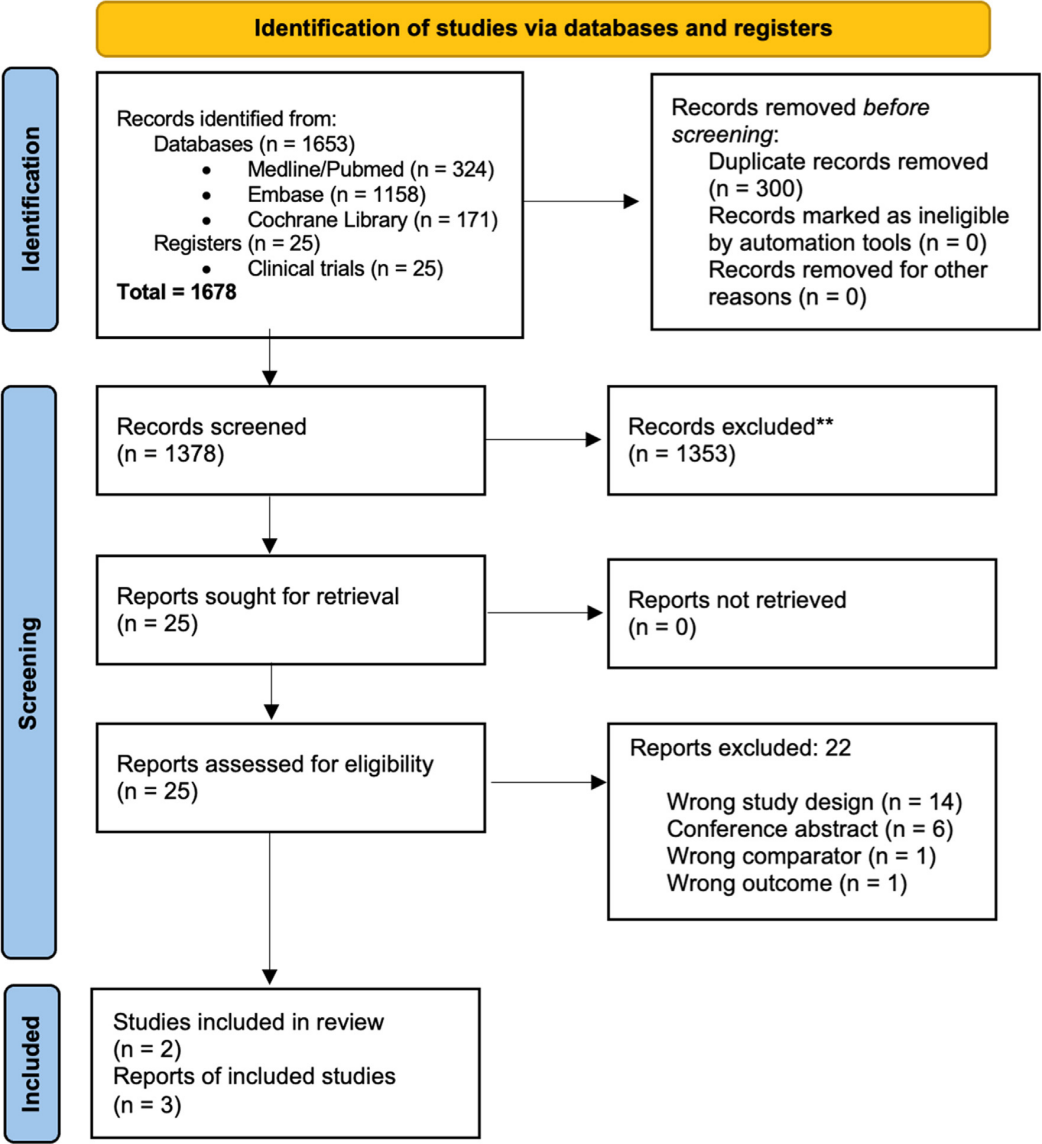
PRISMA 2020 flow diagram of study selection.

Of the 1678 records identified, 300 duplicates were removed, and the remaining 1378 records were screened to verify eligibility criteria. Of these, 1353 records were excluded because they did not meet the eligibility criteria, and the full texts of the remaining 25 records were read. Finally, three articles were included from two RCTs, the Flair [18] and E1912 [19,20] studies. The complete selection flowchart is presented in Figure 1.

The characterization of clinical trials included in the systematic review is presented in Table 1.

**Table 1:** Characterization of clinical trials included in the systematic review.

Study	Publication	Participants	Population	Interventions	Male n (%)	Age	Maximum Follow-up
E1912 (NCT02048813)	Shanafelt et al., 2019 [19] and Shanafelt et al., 2022 [20]	Total: 529; IR: 354; FCR: 175	Patients aged ≤70 years with previously untreated CLL or SLL and in need of therapy according to the iwCLL	Experimental: IR Comparator: FCR	Total: 356 (67.3); IR: 236 (66.7); FCR: 120 (68.6)	Mean total: 56.7 ± 7.4; IR: 56.7 ± 7.5; FCR: 56.7 ± 7.2	Median: 5.8 years (70 months)
Flair (ISRCTN01844152 and EudraCT, 2013–001,944–76)	Hillmen et al., 2023 [18]	Total: 771; IR: 386; FCR: 385	Naive CLL or SLL patients considered fit to receive FCR, between 18 and 75 years of age with a WHO performance status of 2 or less and disease status requiring treatment according to iwCLL	Experimental: IR Comparator: FCR	Total: 565 (73); IR: 283 (73); FCR: 282 (73)	Median total: 62 years (IQR 56–67); IR: 63 (IQR 55- 67); FCR: 62 (IQR 56–67)	Median: 4.4 years (53 months; IQR 41–61)

CLL: Chronic lymphocytic leukaemia; SLL: Small lymphocytic lymphoma; IR: Ibrutinib plus rituximab; FCR: Fludarabine, cyclophosphamide and rituximab; IQR: interquartile range; WHO: World Health Organisation; iwCLL: International Workshop on CLL criteria.

The population of the E1912 trial [19,20] consisted of 529 patients recruited in the USA diagnosed with CLL or SLL, previously untreated and in need of therapy according to the iwCLL criteria. The mean age was 56.7 ± 7.4 years, and the majority were male (67.3 %). According to the Rai classification, the disease stage was intermediate risk, I or II (*n* = 281; 53.1 %), and high risk, III or IV (*n* = 228; 43.1 %). The majority of patients were classified as unmutated (*n* = 281/395; 71.1 %) in terms of the *IGHV* mutation status. A significant portion of the overall population (*n* = 436; 82.4 %) underwent testing for *IGHV* mutation status. Among the 436 patients, *IGHV* status was determined in 395, ensuring the accuracy and reliability of the data.

The Flair study [18] included 771 naive CLL or SLL patients recruited in the United Kingdom and considered fit to receive the FCR regimen. Participants were aged between 18 and 75 years with a WHO performance status of 2 or less and disease status requiring treatment according to iwCLL criteria. The mean age was 62 (interquartile range: 56–67) years, and the majority were male (73 %). Regarding the disease stage, according to the Binet classification, the population was progressive A or B (*n* = 423; 55 %) and stage C (*n* = 348; 45 %). Regarding the *IGHV* mutation status, half of the patients were classified as unmutated (*n* = 388; 50 %).

**Figure 2:**
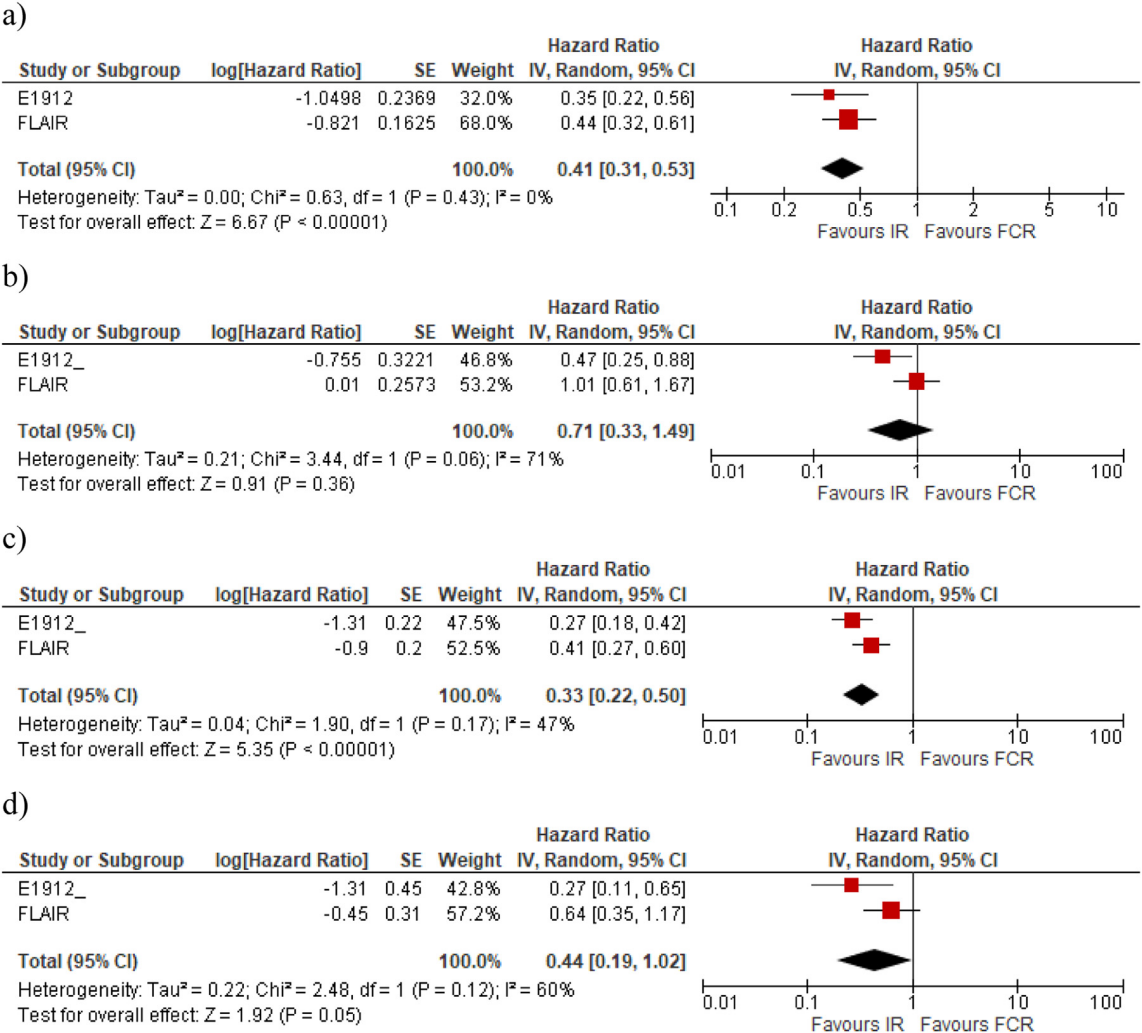
Forest plot of hazard ratio for: a) global PFS; b) global OS; c) PFS in subgroups of with unmutated IGVH; d) PFS in subgroups with mutated IGVH.

The meta-analysis performed with a random model shows that the IR regimen is more effective than FCR for PFS (Tables 2 and 3) with the risk being reduced by 59 % with IR compared to FCR (HR: 0.41; 95 % CI: 0.31–0.53), with moderate certainty of evidence (Figure 2a). However, the mean OS for IR compared with FCR (Figure 2b) was (HR: 0.71; 95 % CI: 0.33–1.49) with very low certainty of evidence, showing no significant difference for this outcome (Tables 2 and 3).

**Table 2:** Results of global outcomes progression-free survival, overall survival andsevere adverse events.

Outcome	Study	Follow up time(years)	Intervention	Participants	Events	Measure of Effect	Measure of Effect by meta-analysis
Global PFS	NCT02048813-E-1219 [20]	5.8	IR	354	84	HR: 0.37; 95 % CI: 0.27–0.51	–
			FCR	175	74		
	NCT02048813-E-1219 [19]	3	IR	354	37	HR: 0.35; 95 % CI: 0.22–0.56	HR: 0.41; 95 % CI: 0.31–0.53
			FCR	175	40		
	FLAIR [18]	4.4	IR	386	59	HR: 0.44; 95 % CI: 0.32–0.60	
			FCR	385	118		
PFS - unmutated IGHV	NCT02048813-E-1219 [20]	5.8	IR	210	56	HR: 0.27; 95 % CI: 0.18 - 0.41	HR: 0.33; 95 % CI: 0.22 - 0.50
			FCR	71	42		
	FLAIR [18]	4.4	IR	194	38	HR: 0.41; 95 % CI: 0.28 - 0.61	
			FCR	194	77		
PFS - mutated IGHV	NCT02048813-E-1219 [20]	5.8	IR	70	44	HR: 0.27; 95 % CI: 0.11 - 0.62	HR: 0.44; 95 % CI: 0.19 - 1.02
			FCR	44	15		
	FLAIR [18]	4.4	IR	146	27	HR: 0.64; 95 % CI: 0.35 - 1.16	
			FCR	148	19		
OS	NCT02048813-E-1219 [19]	3	IR	354	4	HR: 0.17; 95 % CI: 0.05–0.54)	–
			FCR	175	10		
	NCT02048813-E-1219 [20]	5.8	IR	354	21	HR: 0.47; 95 % CI: 0.25–0.89)	HR: 0.71; 95 % CI: 0.33–1.49)
			FCR	175	18		
	FLAIR [18]	4.4	IR	386	31	HR: 1.01; 95 % CI: 0.61–1.68)	
			FCR	385	29		
SAE	NCT02048813-E-1219 [19]	3	IR	352	282	RR = 1.00; 95 % CI: 0.91–1.10); p-value = 0.924	–
			FCR	158	126		
	NCT02048813- E-1219 [20]	5.8	IR	352	257	RR = 0.88; 95 % CI: 0.80–0.97); p-value = 0.015	–
			FCR	158	131		
	FLAIR [18]	4.4	IR	384	205	RR = 0.99; 95 % CI: 0.87–1.13); p-value = 0.93	–
			FCR	378	203

Concerning the *IGHV* mutation, for unmutated CLL patients, the two studies show a benefit of IR compared with FCR in terms of PFS. While in the Flair trial, the difference was significant and precise for unmutated *IGHV*, the difference was not significant in the E1912 trial, even though the mean effect was in favour of IR (Table 2). Figures 2C and 2D present the meta-analysis for PFS in the subgroups without and with *IGVH* mutations, respectively. In the meta-analysis, the PFS was significantly better for IR than for FCR in individuals with unmutated *IGHV* (HR: 0.33; 95 % CI: 0.22–0.50). For those with mutated *IGHV*, the results showed no statistically significant differences (HR: 0.44; 95 % CI: 0.19–1.02).

There were no OS data available for a meta-analysis because only the E1912 trial presented data. This trial found that IR is more effective in reducing the risk of death in patients with unmutated *IGHV* (HR: 0.35; 95 % CI: 0.15–0.80) when compared with FCR. For patients with mutated *IGHV*, the result was not statistically different (HR: 0.72; 95 % CI: 0.15–3.47).

The RR for SAE was 0.88 (95 % CI: 0.80–0.97; p-value = 0.015) in the Flair study, and 0.99 (95 % CI: 0.87–1.13; p-value = 0.93) for the E1912 study; for this outcome, the certainty of evidence was classified as low. The incidence of Grade 3 or higher adverse events were different between the studies. However, both showed less SAE of neutropenia (14 %) and anaemia (3 %) for the IR group compared with the FCR group (54 % and 14 %, respectively). SAEs of interest with the use of ibrutinib, such as hypertension (11.4 % versus 1.9 %) and cardiac event (7.7 % versus 0 %), were reported more frequently in the IR compared to FCR arm of the E1912 study, respectively [[18], [19], [20]]. These results are presented in Table 3.

**Table 3:** Certainty of evidence by Grading of Recommendations Assessment, Development and Evaluation (GRADE) Critical.

Certainty assessment							Events / No. of patients		Effect		Certainty	Importance
No. of studies	Study design	Risk of bias	Inconsistency	Indirectness	Imprecision	Other considerations	IR	FCR	Relative (95 % CI)	Absolute (95 % CI)		
**Progression-free survival (follow-up: median 3 years)**												
2 [18,20]	randomized trials	serious^a^	not serious	not serious	not serious	none	96/740 13.0 %	158/560 28.2 %	**HR 0.41** (0.31–0.53)	313 more per 1000 (from 229 more to 393 more)	⨁⨁⨁◯ Moderate^a^	Critical
**Overall survival (OS) (follow-up: range 4.4 years to 5.8 years)**												
2 [18,20]	randomized trials	serious^a^	serious^b^	not serious	serious^c^	none	52/740 7.0 %	47/560 8.4 %	**HR 0.71** (0.33–1.49)	81 more per 1000 (from 51 fewer to 345 more)	⨁◯◯◯ Very low^a,b,c^	Critical
**Severe adverse events - Grades 3–4 (follow-up: range 4.4 years to 5,8 years)**												
2 [18,20]	randomized trials	serious^d^	not serious	not serious	serious^e^	none	**E1912 - Shanafelt et al., 2022:** RR: 0.88; 95 % CI: 0.80–0.96 **FLAIR - Hillmen et al., 2023:** RR: 0.99; 95 % CI: 0.87–1.13	⨁⨁◯◯ Low^d,e^	Critical			

IR: ibrutinib and rituximab; FCR: fludarabine, cyclophosphamide and rituximab; CI: confidence interval; HR: hazard ratio.

Explanations:.

a. According to the assessment performed using the ROB-2 tool, the two RCTs had some concerns regarding the overall risk of bias, both for the progression-free survival and for overall survival outcomes. The limitations of the studies are related to the randomization process since neither study reported allocation concealment.

b. Considering a clinically important difference in the threshold of 0.85, the point estimates of the studies are located on opposite sides (Hillmen 2023 [18] - HR: 1.01 and Shanafelt 2022 [20] - HR: 0.47), indicating an inconsistency in the studies' results for the overall survival.

c. Considering a clinically important difference threshold of 0.85, the summary estimate of the meta-analysis of the overall survival outcome crossed this threshold and the null effect line, indicating an imprecision in the results.

d. The two RCTs were classified as having some concerns regarding the overall risk of bias according to the assessment performed using the ROB-2 tool in respect to severe adverse events. The limitation of the studies is related to the randomization process and the deviation from the intended interventions domains since neither study reported allocation concealment and did not perform intention-to-treat analysis.

e. Considering the clinically important difference threshold of 0.85, the 95 % confidence interval of Shanafelt crossed this threshold, and the 95 % confidence interval of the Hillmen study crossed the null effect line, indicating that the studies have imprecision regarding their results.

**Figure 3:**
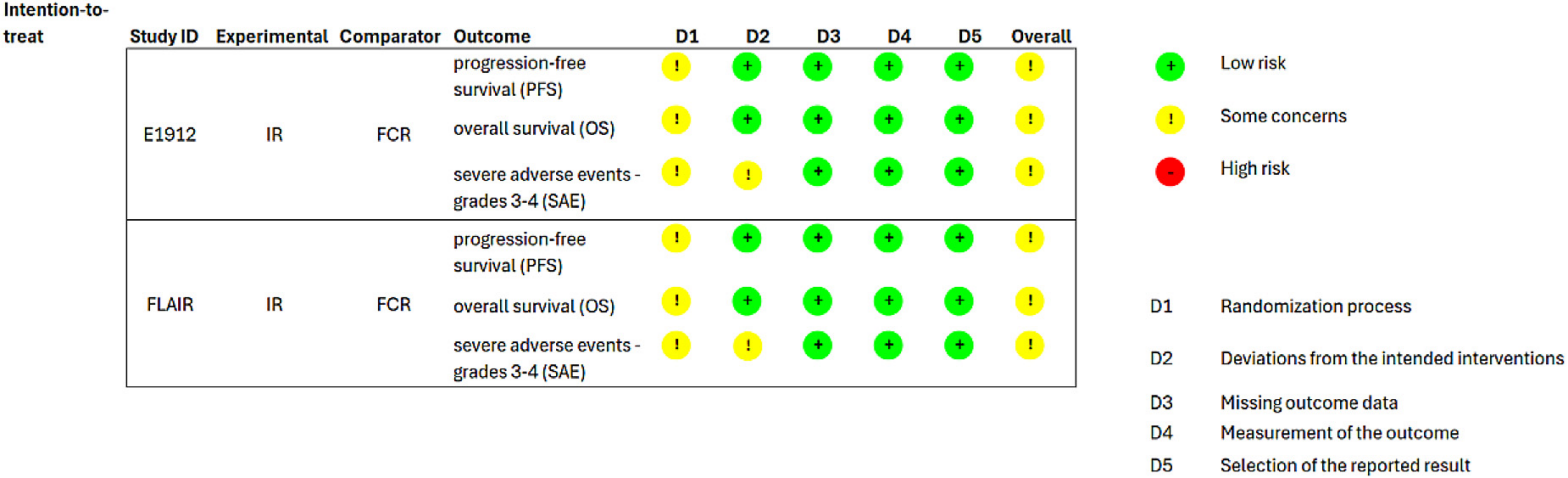
Risk of bias summary.

The global risk of bias was evaluated, as there was some concern regarding the lack of concealed allocation in outcomes of both RCTs. Additionally, for SAE, the analysis was not by intention-to-treat (Figure 3). The GRADE certainty of evidence was evaluated as moderate for PFS because of the risk of bias; very low for OS because of the risk of bias, inconsistency and imprecision; and low for SAE because of the risk of bias and imprecision (Figure 3).

## Discussion

This systematic review showed a clinically relevant effect of IR on PFS compared to using FCR as first-line treatment in patients. Furthermore, the results indicate no difference in relation to the OS and SAE. The European Society for Medical Oncology created a scale to assess the magnitude of the clinical benefit of relevant outcomes for oncology, and when considering only the relative benefit observed as the HR, it is possible to affirm that a lower limit of the HR confidence interval (0.31) of IR reached the threshold of clinical benefit (≤0.70) for PFS when compared to the FCR regimen. Although the OS did not show a statistical difference between the interventions, it is possible to observe that the lower limit of the HR 95 % CI also reached the threshold of clinical benefit (≤0.70 for control >12 months) using the IR intervention, that is, a lower limit of 0.33 [21].

A study conducted to assess the preferences of CLL patients demonstrated that the most important outcome of treatment for them would be increased PFS. In addition, the study indicates a preference for using daily oral medications compared to intravenous medications. In view of this, one study emphasizes the importance of the systematic review findings concerning increased PFS and the use of orally administered ibrutinib [22].

Although ibrutinib and rituximab have been drugs with known efficacy and safety for the treatment of CLL for some years, the first clinical trial, ALLIANCE, that studied the combination of these two drugs in CLL was only published in 2018 [23]. In this study, the IR regimen compared to ibrutinib monotherapy and bendamustine plus rituximab (BR) showed no significant difference between IR and ibrutinib monotherapy regarding PFS in the treatment of older CLL patients [23]. Thus, this was the only study on the IR regimen used to treat CLL available for inclusion in the two systematic reviews with network meta-analysis published until 2021 [24,25]. As it employed the network meta-analysis methodology, the amount of data is quite relevant to the study results; it is desirable to have the most significant number of clinical trials of evaluated interventions.

Another analysis based on indirect comparisons included the data from the publication of the long-term results of the E1912 study in 2022 [20], and, for the first time, data from the comparison between IR and FCR in CLL treatment could be assessed [26]. This analysis demonstrated that there was no significant difference between IR (data from the ALLIANCE [23] and E1912 [20] studies) and ibrutinib plus obinutuzumab concerning PFS. Furthermore, these combinations were quite similar to ibrutinib monotherapy and venetoclax plus obinutuzumab. Together, these therapeutic options were better than FCR, BR, chlorambucil plus obinutuzumab and chlorambucil monotherapy, in decreasing order of PFS results [26]. However, due to the publication date, the analysis of the indirect comparison study did not include the FLAIR trial [18] (published in 2023), which would provide more data on the comparison between IR and FCR and potentially provide information with a higher level of certainty.

In this context, the clinical trial data from the comparison between IR and FCR regimens are highly relevant for healthcare decision-makers, highlighting the need to identify the best and most current evidence of efficacy and safety for this comparison. To our knowledge, the present study is the first systematic review to evaluate the efficacy and safety of IR for the first-line treatment of CLL patients compared to FCR. This systematic review demonstrated that IR is more effective than FCR in terms of PFS. However, the results, show no statistically significant difference between IR and FCR regarding OS. Unlike previous systematic reviews, an additional trial (FLAIR [18]) was included. In addition to providing more information about IR and increasing the statistical power of the clinical results, adding the FLAIR trial allowed the possibility to compare the IR with FCR regimens in another healthcare setting since the trial was conducted in the United Kingdom.

This study reduced the certainty of evidence in all outcomes evaluated mainly because of the potential risk of bias due to the randomization process since neither study reported allocation concealment. Besides that, this current study has limitations that should be highlighted. Some differences between the baseline characteristics of the populations of both clinical trials and their follow-ups may have contributed to inevitable heterogeneity in the results. First, the fact that E1912 [19,20] was conducted in the United States and FLAIR [18] in the United Kingdom requires us to consider potential differences in the healthcare systems and clinical protocols of each country. These differences in the context of the clinical trials may be related to the baseline characteristics and prognosis of CLL patients. For example, patients were staged using the Rai system in E1912 [19] and the Binet system in FLAIR [18], which are two staging systems that employ distinct stages and criteria definitions. Regarding *IGHV* status, 28.9 % of patients had a mutation in E1912 [19] while 38 % were mutated in FLAIR [18]. A difference in the ages of patients in clinical trials was also observed. In E1912 [19], the average age was 56.7 years, in both groups. In FLAIR [18], on the other hand, the median age was 62 years; 63 years in the subgroup with IR and 62 years with FCR. Concerning the proportion of patients in the IR arms of the two studies, E1912 [19] had 66.92 % (354/529) and FLAIR [18] 50.06 % (386/771). Furthermore, the difference in follow-up between clinical trials may also have contributed to some heterogeneity in the results. While patients were followed for 70 months (median) in E1912 [20], the follow-up was 53 months in FLAIR [18].

As mentioned in the results of this study, the follow-up and *IGHV* status were mainly related to the divergence on whether IR was favoured in comparison to FCR. The benefit of IR seems to be greater for the *IGHV* group, but this needs to be confirmed with more clinical data. Thus, the difficulty in weighing these differences between studies became another important limitation of the study. To minimize these differences, a meta-analysis of the results is presented, prioritizing data from the global population without stratification for OS by *IGHV* status. On the other hand, it was impossible to pool the safety data (SAE) in a meta-analysis due to the differences in follow-up times. In any case, we consider that this lack of a safety meta-analysis did not hinder the interpretation of the effects of IR.

## Conclusion

Regarding PFS, IR was more effective than FCR in the first-line treatment of CLL. On the other hand, no additional OS or SAE benefits of IR were observed compared to FCR. Regarding safety, IR was shown to be at least as safe as FCR. Despite some concerns about heterogeneity observed between clinical trials and the certainty of evidence assessed, the results of this systematic review indicate that ibrutinib with rituximab should be considered an effective and safe regimen in the first-line treatment of CLL.

## Funding

No funding.

## Data availability statement

The data that support the findings of this study are available from the corresponding author upon reasonable request.

## Conflicts of interest

The authors declare no conflicts of interest.

## Appendix Supplementary materials

Image, application 1

Image, application 2

## Footnotes

Supplementary material associated with this article can be found in the online version at doi:10.1016/j.htct.2025.106234.
