# Supplementary material for: Efficacy and safety analysis of the use of ibrutinib associated with rituximab for the first-line treatment of patients with chronic lymphocytic leukaemia
Source: Hematol Transfus Cell Ther. 2025 Dec 15;48(1):106234. doi: 10.1016/j.htct.2025.106234 (PMC12769392; doi:10.1016/j.htct.2025.106234)
Supplement: Supplementary file 1 [file mmc1.docx]

# **Supplementary Material**

**Table S1 – PICOS**

| **Patient** | Patients with chronic lymphocytic leukemia recently diagnosed in first-line treatment |
| --- | --- |
| **Intervention** | Ibrutinib + rituximab (IR) |
| **Comparator** | Fludarabine + cyclophosphamide + rituximab (FCR) |
| **Outcomes** | Progression-free survival (PFS), overall survival (OS), severe adverse events - Grades 3-4 (SAE) or quality of life (QOL) |
| **Study** | Randomized controlled trials |

**Table S2 – Research strategy**

| **Database (search date)** | **Search strategy** | **Number of records** |
| --- | --- | --- |
| MEDLINE via PubMed (December 14, 2023) | (("Leukemia, Lymphocytic, Chronic, B-Cell"[Mesh]) OR (((((((Chronic Lymphocytic Leukemia) OR (Small Cell Lymphoma)) OR (Chronic Lymphatic Leukemia)) OR (Small Lymphocytic Lymphoma*)) OR (CLL Lymphoplasmacytoid Lymphoma*)) OR (Chronic Lymph* Leukemia*)) OR (Lymphocytic Lymphoma*))) AND ((("ibrutinib" [Supplementary Concept]) OR ((Ibrutinib) OR (Imbruvica))) AND (("Rituximab"[Mesh]) OR (((Rituximab) OR (Rituxan)) OR (Mabthera)))) | 324 |
| EMBASE (December 14, 2023) | ('lymphatic leukemia'/exp OR 'chronic lymph* leukemia*' OR 'lympho* leukaemia' OR 'small cell lymphoma' OR 'small lymphocytic lymphoma*' OR 'cll lymphoplasmacytoid lymphoma*') AND ('ibrutinib'/exp OR 'ibrutinib' OR 'imbruvica') AND ('rituximab'/exp OR 'blitzima' OR 'cimabior' OR 'mabthera' OR 'redditux' OR 'reditux' OR 'retuxira' OR 'ristova' OR 'ritemvia' OR 'ritucad':ti,ab,kw OR 'ritumax' OR 'rituxan' OR 'rituximab' OR 'rituxin' OR 'rituzena' OR 'rixathon' OR 'riximyo') AND [embase]/lim NOT ([embase]/lim AND [medline]/lim) | 1,158 |
| Cochrane Library (December 14, 2023) | #1 MeSH descriptor: [Leukemia, Lymphocytic, Chronic, B-Cell] explode all trees  #2 ("chronic lymphocytic leukemia") OR (Small Cell Lymphoma) OR (chronic lymph* leukemia*) OR (Small Lymphocytic Lymphoma*) OR (CLL Lymphoplasmacytoid Lymphoma*) (Word variations have been searched)  #3 #1 OR #2  #4 (Ibrutinib) OR (Imbruvica) (Word variations have been searched)  #5 MeSH descriptor: [Rituximab] explode all trees  #6 (Rituximab) OR (Rituxan) OR (Mabthera) OR (Riximyo) (Word variations have been searched)  #7 #5 OR #6  #8 #4 AND #6  #9 #3 AND #8 | 171 |
| ClinicalTrials.gov (1 December 14, 2023) | (Chronic Lymphocytic Leukemia OR leukemia b cell OR Leukemia lymphocytic chronic OR Lymphocytic Lymphoma OR Small Lymphocytic Lymphoma OR B Cell Chronic Lymphocytic Leukemia OR lymphocytic leukemia chronic) AND (Ibrutinib AND Rituximab) | 25 |
| TOTAL | | 1,678 |

**Supplement 2 – list of studies excluded in the full-text reading selection phase**
