# Supplementary material for: Efficacy and safety analysis of the use of ibrutinib associated with rituximab for the first-line treatment of patients with chronic lymphocytic leukaemia
Source: Hematol Transfus Cell Ther. 2025 Dec 15;48(1):106234. doi: 10.1016/j.htct.2025.106234 (PMC12769392; doi:10.1016/j.htct.2025.106234)
Supplement: Supplementary file 2 [file mmc2.docx]

Supplement 2 – list of studies excluded in the full-text reading selection phase

(27) M, Bal V, Cascella T, Shah A, Nanavaty M, Gala S. Impact of dose-difference in comparator arms among clinical trials on treatment effect size in a network meta-analysis: A case of first-line therapies in chronic lymphocytic leukemia (CLL). Blood [Internet]. 2016;128(22). Available from: https://www.embase.com/search/results?subaction=viewrecord&id=L614266693&from=export. 

(28) Xu Y, Fahrbach K, Dorman E, Van S, en S, Diels J, et al. A bayesian network meta-analysis (NMA) of therapies for treatment-naive chronic lymphocytic leukemia (TN-cll) patients ineligible for full-dose fludarabine therapy. Value in Health. 2017;20(5):A89. 

Woyach JA, Ruppert AS, Heerema NA, Zhao W, Booth AM, Ding W, et al. Ibrutinib Regimens versus Chemoimmunotherapy in Older Patients with Untreated CLL. New England journal of medicine. 2018;379(26):2517‐2528. 

(29) Shanafelt TD, Wang V, Kay NE, Hanson CA, O’Brien SM, Barrientos JC, et al. A randomized phase III study of ibrutinib (PCI-32765)-based therapy vs. standard fludarabine, cyclophosphamide, and rituximab (FCR) chemoimmunotherapy in untreated younger patients with chronic lymphocytic leukemia (CLL): A trial of the ECOG-ACRIN cancer research group (E1912). Blood [Internet]. 2018;132. Available from: https://www.embase.com/search/results?subaction=viewrecord&id=L626386785&from=export. [http://dx.doi.org/10.1182/blood-2018-120779](http://dx.doi.org/10.1182/blood-2018-120779)

(30) Ball S, Vutthikraivit W, Das A, Edwards PJ, Hardwicke F, Short NJ, et al. Risk of infection with ibrutinib in patients with b-cell malignancies: A meta-analysis of phase iii randomized controlled trials. Blood [Internet]. 2018;132. Available from: https://www.embase.com/search/results?subaction=viewrecord&id=L626530500&from=export [http://dx.doi.org/10.1182/blood-2018-99-113743](http://dx.doi.org/10.1182/blood-2018-99-113743).

(31) Sardar M, Malik SU, Khan AY, Sohail CS, Ahmad MQ, Amin SN, et al. Role of ibrutinib based regimen in chronic lymphocytic leukemia. Blood [Internet]. 2018;132. Available from: https://www.embase.com/search/results?subaction=viewrecord&id=L626479653&from=export http://dx.doi.org/10.1182/blood-2018-99-110257 

(32) Sardar M, Malik SU, Khan A, Idrees M, Ahmad Q, Sohail C, et al. Efficacy of Ibrutinib-Based Regimen in Chronic Lymphocytic Leukemia: A Systematic Review. J Hematol. 2019;8(1):1–10. 

(33) Shanafelt TD, Wang V, Kay NE, Hanson CA, O’Brien SM, Barrientos JC, et al. Ibrutinib and Rituximab Provides Superior Clinical Outcome Compared to FCR in Younger Patients with Chronic Lymphocytic Leukemia (CLL): Extended Follow-up from the E1912 Trial. Blood. 2019;134:33.

(34) Adhikari N, Zaw MH, Swarup S, Sultan A, Sharma U, Thi WP, et al. Risk of atrial fibrillation and pulmonary toxicities in patients with chronic lymphocytic leukemia or small lymphocytic lymphoma treated with ibrutinib. JNCCN Journal of the National Comprehensive Cancer Network [Internet]. 2019;17(3). Available from: https://www.embase.com/search/results?subaction=viewrecord&id=L629676794&from=export http://dx.doi.org/10.6004/jnccn.2018.7158. 

(35) Davids MS, Waweru C, Le Nouveau P, Padhiar A, Singh G, Abhyankar S, et al. Comparative Efficacy of Acalabrutinib in Frontline Treatment of Chronic Lymphocytic Leukemia: A Systematic Review and Network Meta-analysis. Clin Ther. 2020;42(10):1955-1974.e15. 

(36) Haddad PA, Ganey N, Gallagher KM. Comparative Efficacy of First-Line Chemotherapy-Free Combinations in Chronic Lymphocytic Leukemia (CLL): A Network Meta-Analysis. Blood. 2020;136:25–6. 

(37) Lee CH, Chen PH, Lin C, Wang CY, Ho CL. A network meta-analysis of maintenance therapy in chronic lymphocytic leukemia. PLoS One. 2020;15(1):e0226879. 

(38) Sudhapalli P, Piena M, Palaka A, Mato A, Van De Wetering G, Manzoor B, et al. Systematic literature review and network meta-analysis comparing therapies for treatmentnaïve patients with chronic lymphocytic leukemia. HemaSphere. 2020;4:320. 

(39) Alrawashdh N, Persky DO, McBride A, Sweasy J, Erstad B, Abraham I. Comparative Efficacy of First-Line Treatments of Chronic Lymphocytic Leukemia: Network Meta-Analyses of Survival Curves. Clin Lymphoma Myeloma Leuk. 2021;21(11):e820–31. 

(40) Wang XV, Hanson CA, Tschumper RC, Lesnick CE, Braggio E, Paietta EM, et al. Measurable residual disease does not preclude prolonged progression-free survival in CLL treated with ibrutinib. Blood. 2021;138(26):2810–27. 

(41) Pophali PA, Wang XV, Zhao F, Kay NE, Hanson CA, Thomas ML, et al. Quality of Life in Patients <=70 Years of Age with Chronic Lymphocytic Leukemia Treated Frontline with Ibrutinib-Rituximab Versus Fludarabine Cyclophosphamide Rituximab: Analysis from ECOG-ACRIN E1912. Blood. 2021;138:1562.

(42) Rawstron A, Webster N, Pitchford A, Dalal S, Bloor A, De Tute R, et al. Depletion And Recovery Of Normal B-Cells During And After Treatment With Chemoimmunotherapy, Ibrutinib Or Venetoclax. HemaSphere. 2022;6:1096–7. 

(43) Caridà G, Ciliberto D, Pelaia G, Staropoli N, Siciliano MA, Cal, et al. FRONTLINE Therapy CLL Without 17p del/P53 Aberrations: Systematic Review And Bayesian Network Meta-Analysis. HemaSphere. 2022;6:1030–1.

(44) Pearson LK, Lalla M, Tracy P, Bannuru RR, Klein AK. Systematic Review and Network Meta-Analysis of Frontline Targeted Therapy in Chronic Lymphocytic Leukemia Shows Comparable Efficacy but Distinct Toxicity. Blood. 2022;140:12423–4. 

(45) Vassilopoulos S, Shehadeh F, Kalligeros M, Tran QL, Schiffman F, Mylonakis E. Targeted therapies in CLL/SLL and the cumulative incidence of infection: A systematic review and meta-analysis. Front Pharmacol. 2022;13:989830. 

(46) Nguyen TT, Nhu NT, Tran VK, Nguyen TTH, Lin CF. Efficacy and Safety of Bruton Tyrosine Kinase Inhibitor Monotherapy Compared with Combination Therapy for Chronic Lymphocytic Leukemia and Small Lymphocytic Lymphoma: A Systematic Review and Meta-Analysis. Cancers [Internet]. 2023;15(7). Available from: https://www.embase.com/search/results?subaction=viewrecord&id=L2022558444&from=export http://dx.doi.org/10.3390/cancers15071996. 

(47) Messori A. Long-term progression-free survival in patients with chronic lymphocytic leukemia treated with novel agents: An analysis based on indirect comparisons. Eur J Haematol. 2023;110(1):60–6.
